# Cr(VI) Adsorption on Red Mud Modified by Lanthanum: Performance, Kinetics and Mechanisms

**DOI:** 10.1371/journal.pone.0161780

**Published:** 2016-09-22

**Authors:** You-Wei Cui, Jie Li, Zhao-Fu Du, Yong-Zhen Peng

**Affiliations:** 1 Beijing University of Technology, College of Energy and Environmental Engineering, Chaoyang District, Beijing, China; 2 Central Iron and Steel Research Institute, Haidian District, Beijing, China; Southwest University, CHINA

## Abstract

Water pollution caused by the highly toxic metal hexavalent chromium **(**Cr(VI)) creates significant human health and ecological risks. In this study, a novel adsorbent was used to treat Cr(VI)-containing wastewater; the adsorbent was prepared using red mud (RM) generated from the alumina production industry and the rare earth element lanthanum. This study explored adsorption performance, kinetics, and mechanisms. Results showed that the adsorption kinetics of the RM modified by lanthanum (La-RM), followed the pseudo-second-order model, with a rapid adsorption rate. Cr(VI) adsorption was positively associated with the absorbent dose, pH, temperature, and initial Cr(VI) concentration; coexisting anions had little impact. The maximum Cr(VI) adsorption capacity was 17.35 mg/g. Cr(VI) adsorption on La-RM was a mono-layer adsorption pattern, following the Langmuir isotherm model. Thermodynamic parameters showed the adsorption was spontaneous and endothermic. The adsorption of Cr(VI) on La-RM occurred as a result of LaOCl formation on the RM surface, which in turn further reacted with Cr(VI) in the wastewater. This study highlighted a method for converting industrial waste into a valuable material for wastewater treatment. The novel absorbent could be used as a potential adsorbent for treating Cr(VI)-contaminating wastewater, due to its cost-effectiveness and high adsorption capability.

## Introduction

Rapid economic development has created significant environmental pollution in China, with heavy metal pollution becoming one of the most significant problems. Hexavalent chromium (Cr(VI)), a typical heavy metal, is one of the 25 most hazardous substances in the priority list of hazardous substances [1]. Cr(VI)-contaminated wastewater is generated in large volumes by many industries, such as leather tanning, plating, electroplating, anodizing baths, rinse waters, and other industrial activities [2]. Survey data indicate there are more than 10,000 electroplating factories in China, collectively discharging more than 4 billion cubic meters of Cr-contaminated wastewater annually [3]. Cr(VI) has a wide range of adverse impacts on plants, animals, and humans. Cr(VI) toxicity can lead to the function failure of liver, lung, kidney, and liver organ, as well as gastric damage in humans, even at low concentrations [4]. Therefore, the U.S. environmental protection agency (EPA) has set the strict limits on Cr(VI) effluent levels, with less than 0.05 mg/L allowed in drinking water.

Many techniques, including of chemical precipitation, membrane filtration, adsorption, ion exchange, electrodialysis, and biological methods, have been explored for their ability to remove Cr(VI) from the contaminated water [5–7]. Among these techniques, adsorption has attracted many interests because of its effectiveness in advanced wastewater treatment processes. It has the advantages of being a simple process, with a low amount of toxic sludge production [8, 9]. Adsorbent is considered to be the most important for an adsorption technology, because it directly influences adsorption capacity and treatment cost. Developing adsorbents with high absorption capacity and low cost is the significant research interest in the environmental materials field.

Some industrial wastes may be reused as low-cost absorbent materials, due to their cost-effectiveness, large volumes, and availability. Red mud (RM) is made of highly alkaline bauxite residues from aluminum production. Approximately one to two tonnes (dry weight) of RM are generated for every ton of aluminum produced [10]. RM disposal costs account for approximately 5% of aluminum production [11]; these high costs increase financial burden and lead to great waste accumulation. The accumulation of these high alkaline solids leads to significant environmental pollution, including surface water, groundwater, soil, and even atmospheric pollution. Due to the potential environmental risks, RM is listed as a hazardous solid waste in China.

To promote the recycle and reuse of industrial solid waste, researches have focused on the value-added use of RM. Most recently, RM has been applied as an adsorbent to remove pollutants from water. When RM is treated properly with acid, heat, redox, or other methods, it has the capacity to adsorb phosphorus, fluoride, heavy metals, and even some organics from wastewater [12–17]. However, few research has been done on the adsorption of Cr(VI) by RM from wastewater. One study found that RM presented a removal capacity of 0.015 mg/g Cr(VI) from wastewater after it was treated by a combination of acid and heat [18]. Although this study showed that adsorption of Cr(VI) could be achieved by RM, the adsorption capacity needed to enhance.

Lanthanum (La) is a typical rare earth element, defined by the international union of pure and applied chemistry (IUPAC) as a set of seventeen chemical elements in the periodic table with similar characteristics as highly active metals. Research suggests that some adsorbents activated by lanthanum experience a significant improvement in adsorption capacity. For example, the phosphate adsorption capacity of La(OH)_3_-modified exfoliated vermiculite (EV) was approximately one order of magnitude higher than the capacity of EV alone [19]. Lanthanum chitosan adsorbents remove fluoride from water effectively, exceeding the adsorption rates of bare chitosan and chitin [20]. Lanthanum is one of the most abundant rare earth. Due to its high reserves and limited uses, La currently has a low price compared with other rare earth in China. As such, increasing its application value would increase its trading price.

In this study, we prepared a novel adsorbent by modifying industrial waste RM using La (this material is referred to La-RM). We then evaluated its performance and kinetics in removing Cr(VI) from aqueous media. The influence of several key parameters and the competitive effects of common co-existing ions were investigated using batch experiments. Emphasis was placed on the adsorption mechanism, by establishing the thermodynamics, the adsorption isotherm, and the adsorption reaction.

## Materials and Methods

### La-RM Preparation

The RM used in the study was collected from Shandong aluminum corporation, China. The main chemical composition of RM included: Al_2_O_3_ (27.16%), SiO_2_ (26.70%), CaO (24.68%), TiO_2_ (8.32%), Na_2_O (7.50%), Fe_2_O_3_ (7.00%), K_2_O (2.54%), MgO (1.07%). Due to different production processes, the RM used in this study had lower Fe_2_O_3_ content and slightly higher TiO_2_ content compared to levels found in other studies [21–23].

The raw RM was first crushed and dried in an oven (DHG-9240A, Shanghai) at 105°C for 48 hours. The sample was sieved; particles passing through the 80 mesh were prepared for further use. Each 10 g prepared RM sample was washed with 500 ml doubly distilled water and filtered through a 0.45 μm membrane to wash out adhering impurities. The washed RM was dried overnight at 105°C and again sieved through a 80 mesh sieve.

To continue the preparation, 10 g samples, each with a ratio of 0.6 part lanthanum from LaCl_3_·7H_2_O (chemical pure grade) to 1 part of the sieved RM, were then mixed in a crucible with 30 ml doubly distilled water and fully stirred. The pH was adjusted to 6 using 0.1 M NaOH and 0.1 M HCl; pH was monitored with a pH meter (WTW340i, Germany). The sample was dried at 105°C over 24 hours. The solid was then cooled, crushed, collected, and activated in a muffle furnace (SX_2_-4-10, Tianjin) at a stable temperature of 400°C for 3 h. The resulting product was crushed, washed, dried, and sieved using the same step as before, resulting in La-RM that was ready for further use.

### Leaching metal test

In order to test the material leaching metals, a standard Chinses determination procedure (HJ 557–2009) was taken for La-RM. The prepared La-RM of 100 g was mixed with ultrapure water at a ratio of 1:10 in a 2L extraction flask, which was continuously vibrated in the coaxial shaker bath (7HZ-82, Changzhou) with 200 rpm for 8h at 25°C. Then, the supernatant was sampled and filtered by 0.45μm membrane after 16 hours’ sedimentation. According to the composition of La-RM, the possible metal cations in the filtrate were analyzed.

### Adsorption studies

Batch experiments were used to investigate the Cr(VI) adsorption performance of La-RM. To establish the key operational parameters, we sequentially investigated the La-RM dose, and the effects of temperature, pH, initial Cr(VI) concentration, and contact time. The adsorption experiments were carried out in 150 ml flasks, in which a dose of 2.0–5.0 g/L of La-RM, and 100 ml of K_2_Cr_2_O_7_ with an initial Cr(VI) concentration of 40 mg/L were added. The mixture pH was adjusted to 7, and then the sample was placed in the shaker bath (7HZ-82, Changzhou) to mix at 150 rpm for 3 h at 25°C.

To explore the efficiency of Cr(VI) adsorption, tests investigating temperature ranges from 5~65°C, and pH levels ranging from 2 to 11. Initial Cr(VI) concentrations ranged from 10–140 mg/L to identify the maximum adsorption capacity. To establish the optimal contact time of the adsorption process, samples were collected from the flasks at 5–180 min internals, with initial Cr(VI) concentrations of 10, 40, 70, and 100 mg/L. To study the effect of coexisting anions, solutions of six common ions (F^-^, SO_4_^2-^, CO_3_^2-^, NO_3_^2-^, PO_4_^3-^ and Cl^-^)^,^ with an initial concentration of 40 mg/L, were added to separate samples of 40 mg/L of Cr(VI). All experiments conducted in triplicate and in parallel; the mean value of the three measurements was used as the final result.

### Analytical methods and calculations

Surface morphologies and sample compositions were examined using scanning electron microscopy and energy dispersive spectroscopy (SEM; JSM-6400, JEOL Ltd., ZEISS). The X-ray diffraction (XRD) patterns were identified in the 2θ range of 15–65 using a diffractometer (D/max-RB, Rigaku) with Cu-Ka radiation (λ = 1.54178 Å). The scanning rate was 0.020 s/step. The samples were manually sieved, without any further disposal, to mitigate any effects of the preferred orientation distorting the observed diffraction patterns. Concentrations of Cr(VI) filtered through a 0.45 μm membrane were measured using an ultraviolet spectrophotometer (UV-1200, Shanghai) based on the diphenylcarbohydrazide spectrophotometric method. Eq 1 was used to calculate the amount of Cr(VI) adsorbed per unit mass of adsorbent:

$$q_{e}=\frac{(C_{i}-C_{e})\times \text{V}}{M}$$ (1)

Eq 2 was used to calculate the removal performance of Cr(VI) from wastewater:

$$\text{Cr}(\text{VI})\;\text{Removal}\;(\%)=\frac{C_{i}-C_{e}}{C_{i}}\times 100$$ (2)

In this expression, *q*_e_ is the quantity of Cr(VI) adsorbed (mg/g); *C*_i_ and *C*_e_ are the initial and final Cr(VI) concentrations (mg/L); V is the volume of the Cr(VI) (L); *M* is the mass of La-RM (g).

Kinetic models of pseudo-first-order (Eq 3) and pseudo-second-order (Eq 4) were expressed as:

$$\text{log}(q_{e}-q_{t})=\log q_{e}-\frac{{\text{k}}_{1}}{2.303}t$$ (3)

$$\frac{\text{t}}{q_{t}}=\frac{1}{{\text{k}}_{2}q_{e}{}^{2}}+\frac{1}{q_{e}}t$$ (4)

In these equations, *q*_*e*_ and *q*_*t*_ are the amount of Cr(VI) adsorbed on La-RM, at equilibrium and over a given time (mg/g); *t* is the contact time (min); and k_1_ (1/min) and k_2_ (g/(mg·min)) are the adsorption rate constants for the pseudo-first-order and pseudo-second-order kinetic models.

The Langmuir (Eq 5) and Freundlich (Eq 6) adsorption models were used:

$$\frac{C_{e}}{q_{e}}=\frac{1}{{\text{K}}_{\text{L}}q_{m}}+\frac{C_{e}}{q_{m}}$$ (5)

$$\log q_{e}=\log\;{\text{K}}_{\text{F}}+\frac{1}{\text{n}}\log C_{e}$$ (6)

In these equations, *C*_*e*_ is the concentration of Cr(VI) solution at equilibrium (mg/L); *q*_*e*_ is the corresponding adsorption capacity (mg/g); K_L_ is the constant of Langmuir model related to the free energy of adsorption (L/mg); *q*_*m*_ is the mono-layer adsorption capacity of La-RM (mg/g); K_F_ is the constant of Freundlich model (L/g); 1/n is the heterogeneity factor of the adsorbent related to the adsorption process.

The Gibbs free energy (ΔG°), enthalpy (ΔH°), and entropy (ΔS°) were calculated to evaluate the thermodynamic nature of the adsorption process, using the following equations:

ΔG°=-RTlnK (7)

$$\ln K=\frac{\mathrm{\Delta S}{}^{\circ}}{\text{R}}-\frac{\mathrm{\Delta H}{}^{\circ}}{\text{R}T}$$ (8)

ΔG°=ΔH°−TΔS° (9)

In these expressions, R is the ideal gas constant (8.314J mol^-1^K^-1^); *T* is the temperature (K); and *K* is the sorption equilibrium constant of the adsorption process, calculated between the solid and liquid phases at equilibrium [19].

The grain size of the RM was calculated using Sherrer equation:

D=0.89λ/Bcosθ (10)

In this expression, *D* is the grain size; *B* is the broadening of diffraction line measured at half its maximum intensity; λ = 1.5418 Å is the wavelength of the Cu-k_α_ X-ray; and *θ* is the Bragg angle.

## Results and Discussion

### Leaching metals of La-RM

Leaching metals of prepared La-RM were determined (Table 1). The toxic metal cations in leaching liquid contain Cr, Ni, Pb, Ti, Mn, Zn and Cu. All these toxic metal concentrations were far less than the requirements of the standards for drinking water quality of China (GB 5749–2006). Even the major elements of Ca and Al are also found less than the standard quality values in GB 5749–2006. The leaching La was only 5.984 mg/L, which could be induced by uncombined La in the surface of RM. There are not any available reports indicating La could induce the toxicity to human beings or plants. Therefore, the data of leaching metals showed the safety of using La-RM in the aqueous environment. Li H N et al also reported that cobalt doped red mud was stable and toxicity free when it was used for catalytic ozonation of bezafibrate in wastewater [24]. Thus, La-RM will not cause secondary pollution.

**Table 1:** Leaching metals concentrations comparing with Chinese national drinking water quality standards.

	Mg	Al	Ca	Cu	Cr	Zn	Ni	Pb	Ti	Mn	La
La-RM (mg/L)	0.032	0.138	3.48	0.082	0.003	0.048	0.009	0.003	0.004	0.002	5. 984
GB 5749–2006 (mg/L)	-	0.2	450	1.0	0.05	1.0	0.02	0.01	-	0.1	-

“-” means not required.

### Effects of operational conditions on Cr(VI) adsorption

**Fig 1:**
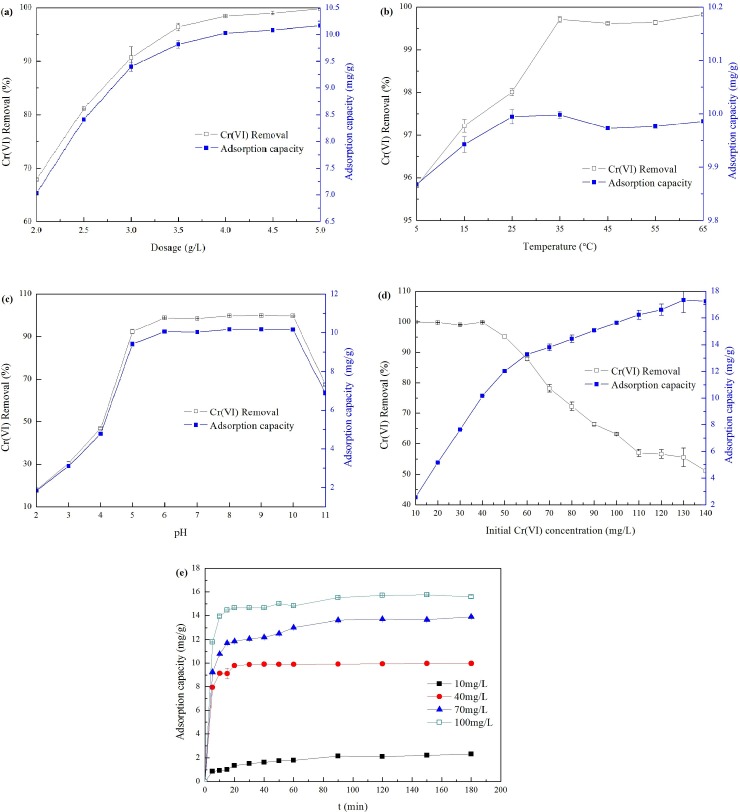
Effects of some operational conditions on Cr(VI) removal and adsorption capacity. (a) The effect of dose (temperature = 25°C, pH = 7, Cr(VI) concentration = 40 mg/L, time = 3 h); (b) The effect of temperature (dosage = 4 g/L, pH = 7, Cr(VI) concentration = 40 mg/L, time = 3 h); (c) The effect of pH (dosage = 4 g/L, temperature = 25°C, Cr(VI) concentration = 40 mg/L, time = 3 h); (d) The effect of initial Cr(VI) concentration (dosage = 4 g/L, temperature = 25°C, pH = 7, time = 3 h); (e) The effect of contact time (dosage = 4 g/L, temperature = 25°C, pH = 7, Cr(VI) concentration = 40 mg/L).

To find the optimal dosage, different amounts of La-RM were tested, ranging from 2.0–5.0 g/L. As Fig 1(A) shows, an increased adsorbent dose leads to increases in Cr(VI) removal efficiency and adsorption capacity. The increased adsorbent had greater surface area and more binding sites available for the Cr(VI) [25]. The smooth curve indicates that the sample was homogeneous. As the La-RM dose increased, the rising rate of Cr(VI) removal decreased. This is due to the split in the flux, or the concentration gradient, between solute concentration in the solution and on the adsorbent’s surface [26]. When the La-RM dose was 4.0 g/L, adsorption efficiency didn’t increase. As such, the following experiments were conducted at this adsorbent dose.

The effect of temperature usually relates to the endothermic or exothermic nature of the adsorption reaction. Fig 1(B) shows the Cr(VI) adsorption in the temperature range of 5°C to 65°C. The Cr(VI) removal efficiency and adsorption capacity increased as the temperature rose from 5°C to 35°C, indicating endothermic adsorption. At temperature of 5°C, the Cr(VI) removal efficiency was 95.8%. A temperature increase improved the Cr(VI) adsorption capacity, resulting in 99.8% Cr(VI) removal efficiency at 35°C. There was no obvious increase in Cr(VI) removal efficiency and adsorption capacity above 35°C, which may be due to the limited number of active sites on the adsorbent. The wastewater temperature used in many Cr(VI)-producing industries, including leather tanning, plating, electroplating, anodizing baths, and rinse waters, ranges from 5–40°C [2]. This result highlights the wide temperature adaptability of La-RM for adsorbing Cr(VI), offering advantages for further industrial treatment applications.

Fig 1(C) shows the Cr(VI) removal as the pH ranged from 2 to 11. Cr(VI) adsorption was highly efficient at solution pH values ranging 5 to 10. Optimal adsorption conditions occurred at a pH of 9, with Cr(VI) removal exceeding 99%. Cr(VI) adsorption was lower below a pH of 5. The pH dependence of Cr(VI) adsorption is attributed to the pH-dependent equilibria expressed by HCr_2_O_7_^-^, Cr_2_O_7_^2-^ and CrO_4_^2-^ [27]. Cr_2_O_7_^2-^ and HCrO_4_^-^ are the main ions at a pH range of 2–6, while CrO_4_^2-^ dominates at pH values greater than 8. Generally, Cr_2_O_7_^2-^ and CrO_4_^2-^ tend to form substances that are difficult to dissolve in water, but are easily dissolved by acid. This means that some stable structures may form in the La-RM with Cr(VI), but do not exist in a strongly acidic environment. The decrease in Cr(VI) removal when pH was above 10 may be caused by the competitive adsorption of OH^-^. To study the maximum adsorption capacity of La-RM, the following experiments were conducted at a pH of 9.

As Fig 1(D) shows, the initial concentration of Cr(VI) ranged from 10 to 130 mg/L; at these levels, Cr(VI) adsorption increased from 2.57 mg/g to a maximum of 17.35 mg/g. This result shows the effective enhancement of adsorption capability by La, because pure RM has a low Cr(VI) adsorption capability of 0.003 mg/g [18]. At initial concentrations between 10 and 40 mg/L, the linear increase in adsorption capacity was caused by the relative abundance of adsorbent, where all the Cr(VI) can be removed with over 99% removal efficiency. The dynamic trend of the adsorption capacity curve, with initial Cr(VI) concentrations of 40–140 mg/L, was similar to the trend of the adsorption capacity variation with adsorbent dosages (Fig 1(A)). This indicated that the adsorption sites were not abundant when compared to the Cr(VI) concentration. Therefore, although the increased Cr(VI) concentration enhanced adsorption capacity, the Cr(VI) removal efficiency also continuously decreased.

Adsorption experiments were conducted over 180 min to identify the optimum contact time. Fig 1(E) shows the adsorption capacity as a result of different initial concentrations of 10, 40, 70 and 100 mg/L. In the first 10 min, the La-RM experienced fast adsorption kinetics and high Cr(VI) removal. The rapid adsorption during the initial contact time can be attributed to the large number of vacant adsorbent sites and the high solute concentration gradient [28]. As more and more active sites disappeared, slow adsorption rates occurred between 10 and 90 min. The equilibrium point of approximately 90 min occurred in all experiments at initial Cr(VI) concentrations ranging from 10–100 mg/L. Therefore, all adsorption experiments reached equilibrium in 3 hours, similar to other experiments using red mud [29].

### Effect of coexisting ions on adsorption

**Fig 2:**
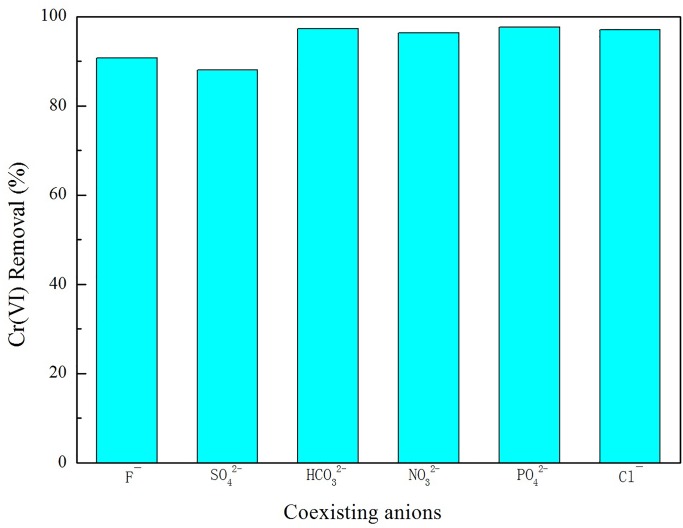
The effect of coexisting anions on Cr(VI) removal.

The effect of different competitive ions, such as F^-^, SO_4_^2-^, CO_3_^2-^, NO_3_^2-^, PO_4_^3-^ and Cl^-^, on Cr(VI) removal was studied at the initial Cr(VI) concentration of 40 mg/L. As Fig 2 shows, the removal efficiency of Cr(VI) decreased to approximately 88% (with SO_4_^2-^) and 91% (with F^-^) compared to the suspension with no additional ions. When studying the other ions, the removal efficiency remained at approximately 97%. SO_4_^2-^ influenced the adsorption capacity of the coexisting anions more than F^-^, which was higher than NO_3_^2-^, Cl^-^, CO_3_^2-^, and PO_4_^3-^.

Although coexisting anions affect the La-RM, Cr(VI) removal remained high, indicating the strong selective adsorption of Cr(VI). In real industrial wastewater, there may be more competitive ions and impurities, such as dyes (with cadmium and zinc), nitrate and sulfate salts, surfactants, sulfide, and formaldehyde in textile industry wastewater [30]; and concentrations of iron, copper, zinc, chromium and nickel with high concentrations of acids, sulfate, and Cl^−^ ions in electroplating wastewater [31]. As such, practical application of La-RM requires further investigation with different types of wastewater.

### Adsorption kinetics

**Fig 3:**
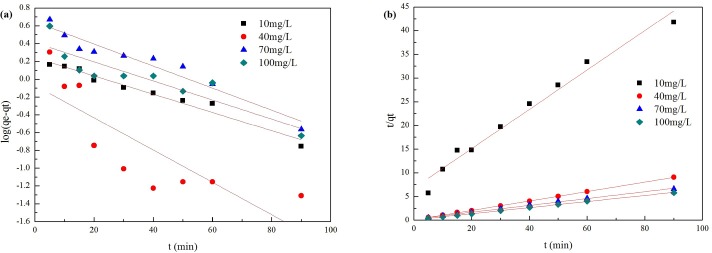
Adsorption kinetics of Cr(VI) on La-RM. (a) Pseudo-first-order kinetic model for Cr(VI) adsorption;(b) Pseudo-second-order kinetic model for Cr(VI) adsorption.

Fig 3 shows the pseudo-first-order (a) plot of log(*q*_e_-*q*_t_) against t and the pseudo-second-order (*b*) plot of *t*/*q*_t_ against t at the different initial Cr(VI) concentrations. Table 2 presents the calculated kinetic parameters including the regression coefficient (R^2^) of pseudo-first-order and pseudo-second-order kinetic models. The adsorption kinetic data recorded by the pseudo-second-order (R^2^≥0.9777) model fit better than the pseudo-first-order model (R^2^≤0.9677) in the initial 10–100 mg/L concentration range. The pseudo-second-order model assumes a mono-layer adsorption system, and that the adsorption mechanism is determined by chemisorption, not physisorption [32]. The maximum adsorption capacities (*q*_m_) in the initial 10–100 mg/L concentration range calculated by this model match the experimental data well (Fig 1(D)).

**Table 2:** The pseudo-first-order and pseudo-second-order kinetic parameters.

Cr(VI)	Pseudo-first-order			Pseudo-second-order model		
	*k*_1_	*q*_m_	R^2^	*k*_2_	*q*_m_	R^2^
10 mg/L	0.0237	1.7495	0.9677	0.0254	2.4078	0.9777
40 mg/L	0.0418	0.8506	0.6794	0.1014	10.0685	0.9998
70 mg/L	0.0285	4.4025	0.9383	0.0201	13.8696	0.9977
100 mg/L	0.0247	2.5694	0.8176	0.0388	15.5788	0.9992

### Adsorption isotherms

**Fig 4:**
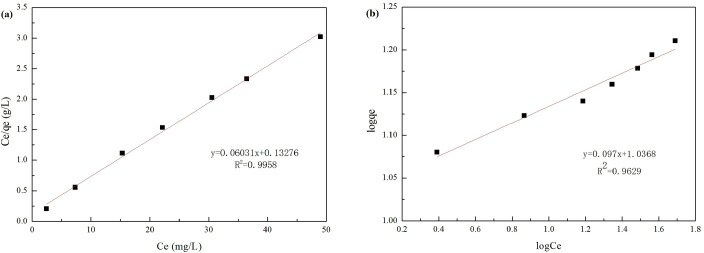
Adsorption isotherms of Cr(VI) on La-RM. (a) Langmuir isotherm model for Cr(VI) adsorption; (b) Freundlich isotherm model for Cr(VI) adsorption.

The equilibrium data were fit to the adsorption isotherms to determine the adsorption capacity and potential for Cr(VI) adsorption. Fig 4 presents the Langmuir plot of *C*_e_ versus *C*_e_/*q*_e_ and the Freundlich plot of log*C*_e_ to log*q*_e_ for the adsorption data. Table 3 shows the relative parameters calculated from the plots. The high regression coefficient 0.9965 of the Langmuir isotherm, compared with the slightly lower 0.9691 of the Freundlich isotherm, suggests that Cr(VI) adsorption on La-RM fit well on the Langmuir isotherm. Previously, Cr(VI) adsorption was also investigated against a Langmuir isotherm using activated carbon prepared from longan seed (LSAC) [33] and magnetic chitosan nanoparticles [34]. This is consistent with our results. In the Langmuir isotherm model, the adsorption process is assumed to be a mono-layer on a uniform surface, with limited adsorption sites [35]. This suggests there is a maximum adsorption capacity, *q*_*m*_, calculated as 16.5810 mg/g. This value is close to the experimental value of 17.35 mg/g. Some novel absorbents were prepared for Cr(VI) removal from liquids in recent years, which showed the different adsorption capacity (Table 4). Comparing with these absorbents, La-RM prepared in this study showed the relatively high adsorption capacity, which indicated the potential prospects for application of Cr(VI) removal from wastewater.

**Table 3:** Langmuir and Freundlich isotherm parameters.

Langmuir			Freundlich		
*q*_m_	*K*_L_	R^2^	n	*K*_f_	R^2^
16.5810	0.4543	0.9965	10.3093	10.8843	0.9691

**Table 4:** Comparison of different adsorbents for Cr(VI) removal.

Adsorbent	Adsorption capacity (mg/g)	Reference
Cetyltrimethylammonium bromide modified red mud	22.20	[36]
Polydopamine/MNP core/shell	9.725	[37]
Activated alumina	7.44	[38]
Bio-char derived from wood chips	1.717	[39]
α-Fe_2_O_3_ nanofibers	16.17	[40]
A carbonaceous material obtained from the diesel engine exhaust mufflers	11.494	[41]
Red Mud modified by lanthanum	17.35	This work

### Thermodynamic study

The thermodynamic parameters were calculated using experimental data (Table 5). The negative ΔG° values indicate that the Cr(VI) adsorption on La-RM is spontaneous. As the temperature rises, the ΔG° value becomes more negative, indicating that the adsorption process becomes less favorable at higher temperatures. This agrees with the variability of Cr(VI) removal at different temperature (referring to Fig 1(B)). The positive ΔH° values suggest endothermic adsorption, consistent with the effect of temperature discussed above. The positive ΔS° revealed the increased disorderliness and randomness at the La-RM solid-solution interface.

**Table 5:** Thermodynamic parameters for Cr(VI) adsorption onto La-RM.

T (°C)	ΔG° (KJ/mol)	ΔS° (J/mol)	ΔH° (KJ/mol)
5	-4.0294	0.1694	43.239
15	-5.1924		
25	-6.1903		
35	-10.7684		
45	-11.0449		
55	-11.5468		
65	-13.9866		

### Surface characteristics of raw RM, La-RM and La-RM absorbed by Cr(VI)

**Fig 5:**
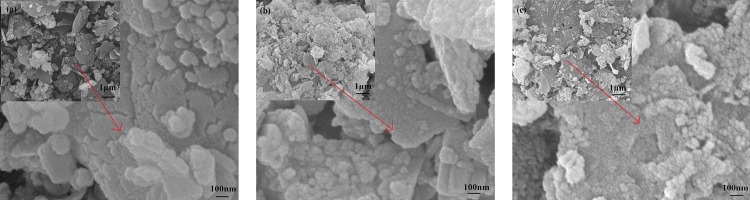
SEM images. (a) SEM image of raw RM; (b) SEM image of La-RM; (c) SEM image of La-RM adsorbed by Cr(VI).

The raw RM powders are composed of luster granules at the micrometer scale with no obvious pores (Fig 5(A)). The raw RM surface became rough after being modified with lanthanum. Many particles accumulate on the surface (Fig 5(B)). Table 6 presents the primary composition of RM and La-RM, as determined by energy dispersive spectroscopy in the top 1 μm of the sample surface. Raw RM was composed of high amounts of Al, Si, and Ca. In La-RM, La content increased from 0 to 28.65% in weight and formed the main element. This demonstrates that the particle accumulation was due to the La. In the La-RM, Ca content decreased significantly, indicating that phase changes may have occurred during La-RM preparation. After adsorbing Cr(VI), the La-RM surface became rougher (Fig 5(C)). The sample surface changes indicated that new phases or crystals might form during the preparation and adsorption process. This deserves further investigation.

**Table 6:** Composition of RM and La-RM

Element	RM		La-RM	
	Wt%	At%	Wt%	At%
** O**	36.32	53.59	23.14	45.68
** Na**	5.55	5.69	0.89	1.22
** Mg**	1.07	1.04	1.2	1.56
** Al**	14.38	12.58	13.66	15.98
** Si**	12.46	10.47	11.23	12.63
** K**	2.11	1.28	2.51	2.03
** Ca**	17.63	10.38	4.32	3.4
** Ti**	4.99	2.46	2.72	1.79
** La**	0	0	28.65	6.51
** Fe**	4.9	2.07	2.28	1.29

### Mechanism of Cr(VI) adsorption on La-RM

**Fig 6:**
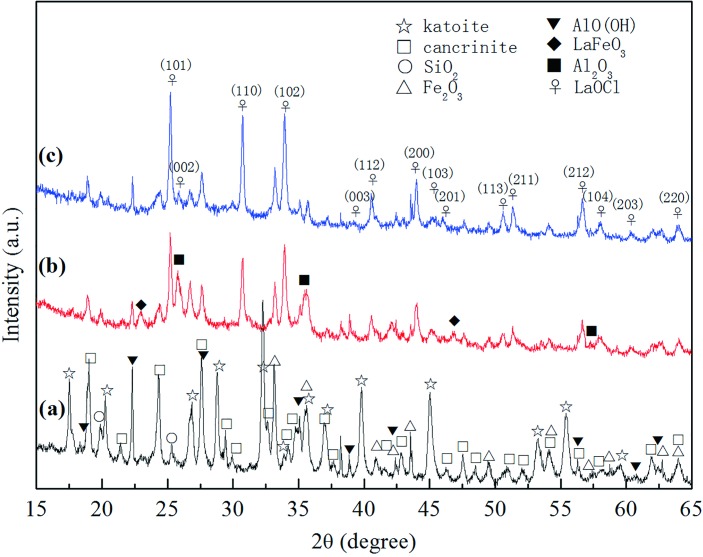
The powder XRD pattern of RM (line a), RM dropped by lanthanum (line b), and La-RM adsorbed by Cr(VI) (line c).

Adsorption data indicated that the La-RM adsorption of Cr(VI) occurred chemically on the surface. As such, the crystal composition was established using XRD (Fig 6). The powder XRD pattern shows that the main phases of the raw RM are katoite and cancrinite, as all the peaks are consistent with the standard references (JCPDS cards PDF#38–0368 and PDF#48–1862). Some peaks correspond to SiO_2_ and Fe_2_O_3,_ which are important components of RM (Table 6). The Al^3+^ exists in the form of AlO(OH), rather than the Al(OH)_3_ found in the raw RM. This may be because Al(OH)_3_ could be annealed under 300°C after losing a H_2_O molecule at a high temperature.

After lanthanum modification, a diffraction pattern for tetragonal LaOCl was present. The peaks of (101) (110) (102) are sharp and clear, consistent with standard JCPDS card 73–2063 of LaOCl. In the meantime, the RM peak was weakened because it was coated by LaOCl; this was observed in the SEM images (Fig 5(B)). No obvious differences were found in the XRD pattern of La-RM after absorbing Cr(VI) from the wastewater compared with La-RM. This indicates that the crystal form transformation occurred after adsorbing Cr(VI). The LaOCl grain size in the La-RM is 25.6 nm. After the La-RM granule absorbed Cr(VI), granule size increased to 38.1 nm. These data suggest that the LaOCl could be responsible for Cr(VI) adsorption from the wastewater.

It has reported that complexation reaction could occur between La(OH)_3_ and PO_4_^2-^ [42,43]. On the same reaction mode, As(VI) in the form of H_2_AsO_4_^-^ and HAsO_4_^2-^ were also found to react with La(OH)_3_ [44]. LaOCl is reported to easily react with H_2_O to form hydrous La(OH)_3_ at pH values greater than 3 [45]. So, it is reasonable to deduce that the same reaction mechanism could occur in our study. These possible reactions of La(OH)_3_ with Cr(VI) radical are dependent on the solution pH (Eqs 12–14) [26]. La(OH)_3_ can exchange the with hydroxy to form different chemical compounds such as ≡La-CrO_4_, ≡La-HCrO_4_ and ≡La-Cr_2_O_4_ with stable valence bond under different pH solutions [46].

$$\text{LaOCl}+{2\text{H}}_{2}\text{O}\to {\text{La}(\text{OH})}_{3}+\text{HCl}$$ (11)

$$\equiv \text{La}-{\left(\text{OH}\right)}_{2}+{\text{CrO}}_{4}^{2-}\to \equiv \text{La}-{\text{CrO}}_{4}+{2\text{OH}}^{-}(\text{pH}>8)$$ (12)

$$\equiv \text{La-OH}+{\text{HCrO}}_{4}^{-}\to \equiv \text{La}-{\text{HCrO}}_{4}+{\text{OH}}^{-}(2<\text{pH}<8)$$ (13)

$$\equiv \text{La}-{\left(\text{OH}\right)}_{2}+{\text{Cr}}_{2}{\text{O}}_{7}^{2-}\to \equiv \text{La}-{\text{Cr}}_{2}{\text{O}}_{7}+{2\text{OH}}^{-}(2<\text{pH}<8)$$ (14)

## Conclusion

This paper described Cr(VI) adsorption using a novel La-RM material; adsorption was positively associated with the La-RM dose, pH, temperature, and initial Cr(VI) concentration. The fast adsorption showed a maximum adsorption capacity of 17.35 mg/g; coexisting ions had little influence on that rate. The spontaneous and endothermic adsorption was a mono-layer system, following the Langmuir isotherm model and pseudo-second-order kinetic model. The La-RM adsorption relied on hydrous La(OH)_3_, which reacted with different forms of Cr(VI) in wide pH ranges in wastewater.

This study demonstrated that the novel La-RM supported Cr(VI) adsorption, providing an excellent way to reuse RM as a valuable material for wastewater treatment. The La modification enhances adoption capability. La is an inexpensive element in rare earth mines; as such, this study also presents a solution that may improve lanthanum application value. To avoid the pollution in the environment, further disposal of the chromium-attached La-RM or the recycle of Cr(VI) from the chromium-attached La-RM should be added as the necessary process for real wastewater treatment. Future research will focus on material regeneration and Cr(VI) recycle to expand engineering application.

## Supporting Information

S1 File Original data of Figs 1–4. (DOC)

## Abbreviations

*q_e_*: Quantity of Cr(VI) adsorbed at equilibrium mg/g
*C_i_*: Initial Cr(VI) concentration mg/L
*C_e_*: Final Cr(VI) concentration mg/L
V: Volume of the Cr(VI) L
*M*: Mass of La-RM g
*q_t_*: Quantity of Cr(VI) adsorbed over a given time mg/g
*t*: Contact time min
k_1_: Adsorption rate constants for the pseudo-first-order kinetic model 1/min
k_2_: Adsorption rate constants for the pseudo-second-order kinetic model g/(mg·min)
K_L_: Constant of Langmuir model L/mg
*q_m_*: Mono-layer adsorption capacity of La-RM mg/g
K_F_: Constant of Freundlich model L/g
n: Heterogeneity factor of Freundlich model
ΔG°: Gibbs free energy KJ/mol
ΔH°: enthalpy KJ/mol
ΔS°: entropy J/mol
R: The ideal gas constant (8.314J mol-1K-1)
*T*: Temperature K
*K*: Sorption equilibrium constant
*D*: Grain size nm
*B*: Broadening of diffraction line measured at half its maximum intensity
λ: Wavelength of X-ray
θ: Bragg angle
R^2^: Regression coefficient
