# Supplementary material for: Cr(VI) Adsorption on Red Mud Modified by Lanthanum: Performance, Kinetics and Mechanisms
Source: PLoS One. 2016 Sep 22;11(9):e0161780. doi: 10.1371/journal.pone.0161780 (PMC5033449; doi:10.1371/journal.pone.0161780)
Supplement: S1 File — (DOC) [file pone.0161780.s001.doc]

**Data of** Fig 1

|  | Removal efficiency (%) | |  | Adsorption capacity(mg/g) | |
| --- | --- | --- | --- | --- | --- |
|  | Average | Y-error |  | Average | Y-error |
| Dose(g/L) |  |  |  |  |  |
| 2 | 67.8818 | 0.20951 |  | 7.0325 | 0.02171 |
| 2.5 | 81.1858 | 0.10476 |  | 8.4108 | 0.01085 |
| 3 | 90.7186 | 2.08444 |  | 9.3983 | 0.08682 |
| 3.5 | 96.4287 | 0.70317 |  | 9.8225 | 0.07163 |
| 4 | 98.453 | 0.04262 |  | 10.0287 | 0.00434 |
| 4.5 | 98.9857 | 0.02131 |  | 10.0829 | 0.00217 |
| 5 | 99.8185 | 0.00852 |  | 10.1677 | 9.00E-04 |
| Temperature(℃) |  |  |  |  |  |
| 5 | 95.8061 | 0.02139 |  | 9.8675 | 0.0021 |
| 15 | 97.2178 | 0.14972 |  | 9.9424 | 0.01472 |
| 25 | 98.0088 | 0.08278 |  | 9.9946 | 0.01323 |
| 35 | 99.7127 | 0.06338 |  | 9.9981 | 0.00651 |
| 45 | 99.6173 | 0.02103 |  | 9.9728 | 0.0021 |
| 55 | 99.6384 | 0.04205 |  | 9.9767 | 0.00421 |
| 65 | 99.8276 | 0.02103 |  | 9.9861 | 0.0021 |
| pH |  |  |  |  |  |
| 2 | 18.1121 | 0.21308 |  | 1.8449 | 0.02171 |
| 3 | 30.4709 | 0.63925 |  | 3.1038 | 0.06512 |
| 4 | 46.7718 | 0.53271 |  | 4.7643 | 0.05426 |
| 5 | 92.3588 | 1.10803 |  | 9.4079 | 0.11287 |
| 6 | 98.794 | 0.04262 |  | 10.059 | 0.00434 |
| 7 | 98.453 | 0.04262 |  | 10.0287 | 0.00434 |
| 8 | 99.8168 | 0.04262 |  | 10.1676 | 0.00434 |
| 9 | 99.8398 | 0.06392 |  | 10.1699 | 0.00651 |
| 10 | 99.8142 | 0.14916 |  | 10.1521 | 0.01519 |
| 11 | 67.5474 | 0.42617 |  | 6.8805 | 0.04341 |
| Initial Cr(VI) (mg/L) |  |  |  |  |  |
| 10 | 99.9222 | 0.06762 |  | 2.5657 | 0.00174 |
| 20 | 99.7516 | 0.04196 |  | 5.1595 | 0.00217 |
| 30 | 99.0115 | 0.10695 |  | 7.6356 | 0.00825 |
| 40 | 99.8398 | 0.06392 |  | 10.1699 | 0.00651 |
| 50 | 95.1399 | 0 |  | 12.0247 | 0 |
| 60 | 87.8266 | 0.64599 |  | 13.2792 | 0.09767 |
| 70 | 78.2397 | 1.22264 |  | 13.8001 | 0.24961 |
| 80 | 72.2681 | 1.41212 |  | 14.4404 | 0.28217 |
| 90 | 66.3864 | 0.52551 |  | 15.0807 | 0.11938 |
| 100 | 63.151 | 0.35069 |  | 15.6342 | 0.08682 |
| 110 | 56.984 | 1.1805 |  | 16.2398 | 0.33643 |
| 120 | 56.6146 | 1.43328 |  | 16.6106 | 0.42052 |
| 130 | 55.5406 | 3.02949 |  | 17.3465 | 0.94617 |
| 140 | 51.0776 | 0 |  | 17.2414 | 0 |

|  | 10mg/L |  | 40mg/L |  | 70mg/L |  | 100mg/L |  |
| --- | --- | --- | --- | --- | --- | --- | --- | --- |
| Time (min) | Average | Y-error | Average | Y-error | Average | Y-error | Average | Y-error |
| 0 | 0 | 0 | 0 | 0 | 0 | 0 | 0 | 0 |
| 5 | 0.8709 | 0.06308 | 7.9539 | 0.1577 | 9.2368 | 0.02171 | 11.7956 | 0.01085 |
| 10 | 0.9339 | 0.06658 | 9.1419 | 0.12616 | 10.7928 | 0.01085 | 13.9403 | 0.08682 |
| 15 | 1.0181 | 0.02171 | 9.1209 | 0.44155 | 11.7179 | 0.08682 | 14.4765 | 0.07163 |
| 20 | 1.3545 | 0.06512 | 9.7937 | 0.06308 | 11.8651 | 0.07163 | 14.6552 | 0.05257 |
| 30 | 1.5227 | 0.05426 | 9.8749 | 0.06658 | 12.0754 | 0.00434 | 14.6552 | 0.03855 |
| 40 | 1.6278 | 0.11287 | 9.9134 | 0.05607 | 12.2015 | 0.00217 | 14.6552 | 0.03154 |
| 50 | 1.7539 | 0.00434 | 9.9029 | 0.05257 | 12.5169 | 0.000868206 | 15.0126 | 0.04262 |
| 60 | 1.796 | 0.00434 | 9.9029 | 0.03855 | 13.0216 | 0.03855 | 14.8339 | 0.06392 |
| 90 | 2.1535 | 0.00434 | 9.9239 | 0.03154 | 13.6313 | 0.03154 | 15.5143 | 0.01752 |
| 120 | 2.1049 | 0.00651 | 9.9449 | 0.01752 | 13.7154 | 0.01752 | 15.7035 | 0.02103 |
| 150 | 2.2171 | 0.01519 | 9.9695 | 0.02103 | 13.6734 | 0.02103 | 15.7456 | 0.05426 |
| 180 | 2.3293 | 0.04341 | 9.9729 | 0.01051 | 13.9046 | 0.04341 | 15.5774 | 0.11287 |

**Data of** Fig 2

| coexisting ions | | | | |
| --- | --- | --- | --- | --- |
|  | Removal efficiency (%) | Y-error | Removal efficiency (%) | Y-error |
| F^-^ | 90.7429 | 0 | 8.7679 | 0 |
| SO_4_^2-^ | 88.0274 | 0.0022 | 8.4315 | 0.0022 |
| HCO_3_^2-^ | 97.2702 | 0.00154 | 9.2963 | 0.00154 |
| NO_3_^-^ | 96.3762 | 0.00132 | 9.2416 | 0.00132 |
| PO_4_^3-^ | 97.5782 | 0.000220003 | 9.3257 | 0.000220003 |
| Cl^-^ | 97.0174 | 0.00022245 | 9.1701 | 0.00022245 |

**Data of** Fig 3

| Adsorption kinetics | | | | | | | | |
| --- | --- | --- | --- | --- | --- | --- | --- | --- |
|  | 10mg/L |  | 40mg/L |  | 70mg/L |  | 100mg/L |  |
| pseudo-first-order | t | log(*q*_e_-q_t_) | t | log(*q*_e_-*q*_t_) | t | log(*q*_e_-*q*_t_) | t | log(*q*_e_-*q*_t_) |
|  | 5 | 0.16387 | 5 | 0.30515 | 5 | 0.66911 | 5 | 0.59659 |
|  | 10 | 0.14466 | 10 | -0.08035 | 10 | 0.49302 | 10 | 0.25655 |
|  | 15 | 0.11766 | 15 | -0.0695 | 15 | 0.33979 | 15 | 0.10351 |
|  | 20 | -0.0111 | 20 | -0.74646 | 20 | 0.30953 | 20 | 0.03759 |
|  | 30 | -0.09336 | 30 | -1.00824 | 30 | 0.26228 | 30 | 0.03759 |
|  | 40 | -0.15402 | 40 | -1.22494 | 40 | 0.23124 | 40 | 0.03759 |
|  | 50 | -0.24013 | 50 | -1.15436 | 50 | 0.1423 | 50 | -0.13491 |
|  | 60 | -0.27309 | 60 | -1.15436 | 60 | -0.05399 | 60 | -0.04015 |
|  | 90 | -0.75504 | 90 | -1.30927 | 90 | -0.5633 | 90 | -0.63585 |
| pseudo-second-order | 5 | 5.74129 | 5 | 0.62862 | 5 | 0.54131 | 5 | 0.42389 |
|  | 10 | 10.70706 | 10 | 1.09387 | 10 | 0.92655 | 10 | 0.71735 |
|  | 15 | 14.7338 | 15 | 1.64458 | 15 | 1.28009 | 15 | 1.03617 |
|  | 20 | 14.76577 | 20 | 2.04213 | 20 | 1.68561 | 20 | 1.36471 |
|  | 30 | 19.70194 | 30 | 3.03802 | 30 | 2.4844 | 30 | 2.04706 |
|  | 40 | 24.57269 | 40 | 4.03494 | 40 | 3.27828 | 40 | 2.72941 |
|  | 50 | 28.5066 | 50 | 5.04903 | 50 | 3.99459 | 50 | 3.33053 |
|  | 60 | 33.40698 | 60 | 6.05884 | 60 | 4.60775 | 60 | 4.04479 |
|  | 90 | 41.79291 | 90 | 9.069 | 90 | 6.60245 | 90 | 5.8011 |

**Data of Fig 4**

| Adsorption isotherms | | | |
| --- | --- | --- | --- |
| Langmuir | | Feundlich | |
| *C*_e_ | *C*_e_/*q*_e_ | log*C*_e_ | log*q*_e_ |
| 2.45702 | 0.20433 | 0.39041 | 1.08007 |
| 7.36239 | 0.55443 | 0.86702 | 1.12317 |
| 15.34989 | 1.1123 | 1.18611 | 1.13988 |
| 22.16531 | 1.53495 | 1.34567 | 1.15958 |
| 30.5435 | 2.02533 | 1.48492 | 1.17842 |
| 36.49071 | 2.33403 | 1.56218 | 1.19408 |
| 49.03629 | 3.01951 | 1.69052 | 1.21058 |
